# Cerebral venous thrombosis as a rare cause of nausea and vomiting in early pregnancy: Case series in a single referral center and literature review

**DOI:** 10.3389/fneur.2022.912419

**Published:** 2022-09-22

**Authors:** Chuan Wang, Xing Hu, Ka U. Lio, Jianhua Lin, Ning Zhang

**Affiliations:** ^1^Department of Obstetrics and Gynecology, Renji Hospital, Shanghai Jiao Tong University School of Medicine, Shanghai, China; ^2^Department of Radiology, Shanghai Ninth People's Hospital, Affiliated to Shanghai Jiao Tong University School of Medicine, Shanghai, China; ^3^Department of Medicine, Temple University Hospital, Lewis Katz School of Medicine at Temple University, Philadelphia, PA, United States

**Keywords:** cerebral venous thrombosis, early pregnancy, nausea and vomiting, hyperemesis gravidarum, headache

## Abstract

**Objectives:**

Cerebral venous thrombosis (CVT) in early pregnancy is extremely rare and evidence limited to only a few published reports. This study aims to present our experience and summarize the available literature to further elucidate the clinical manifestations, treatment, and outcomes of CVT in early pregnancy.

**Methods:**

A retrospective case series of seven patients diagnosed with CVT in early pregnancy (<12 weeks of gestations) in a tertiary referral center (2018–2021), along with a review of published literature.

**Results:**

All the patients presented with nausea, vomiting, headaches, and neurological symptoms including aphasia (*n* = 5, 71.4%), limb weakness (*n* = 4, 57.1%), seizures (*n* = 2, 28.6%), altered mental status (*n* = 3, 42.9%), and blurred vision (*n* = 2, 28.6%). All the patients were diagnosed with CVT by neuroimaging, which revealed various extents of sinus involvement, with the transverse sinus being the most common site (*n* = 7, 100%) followed by the sigmoid sinus (*n* = 5, 71.4%). All the patients received subcutaneous low-molecular-weight heparin once the diagnosis was confirmed. Two patients with rapid deterioration underwent venous thrombectomy, and one patient subsequently underwent decompressive craniotomy but died despite the above interventions. All the other patients proceeded with induced abortion after stabilization and were discharged on oral anticoagulation for 1 year. On the 12-month follow-up, the MRI/magnetic resonance venography (MRV) revealed recanalization of sinuses and resolution of thrombi.

**Conclusions:**

Cerebral venous thrombosis (CVT) in early pregnancy represents a diagnostic challenge given its rarity and nonspecific overlapping clinical features with nausea and vomiting of pregnancy/hyperemesis gravidarum (NVP/HG), which could lead to delay in diagnosis and result in rapid deterioration. Persistent or aggravating headaches combined with other focalizing neurological symptoms in NVP/HG patients could be an initial sign of CVT. Urgent MRI/MRV remains the cornerstone for diagnosis, and immediate anticoagulation is the key for disease prognosis. Glasgow coma scale (GCS) evaluation on admission is probably correlated with the prognosis. Early pregnancy combined with CVT is not a contraindication of continued pregnancy.

## Introduction

Cerebral venous thrombosis (CVT) is a rare yet life-threatening cerebrovascular event with an estimated incidence of <1.5 per 1,00,000 annually (1). Major risk factors include female sex and prothrombotic conditions either inherited (i.e., thrombophilia) or acquired (i.e., antiphospholipid syndrome, use of oral contraceptives, malignancy, pregnancy, and puerperium) (2). Previous studies showed that CVT accounts for only 2% of pregnancy-related stroke (3) and most often presents in the third trimester of pregnancy and the puerperium because of its hypercoagulable state. CVT in early pregnancy is extremely rare, with evidence limited to only a few published reports (4). The aims of this study were to present our experience and to summarize all available literature to further elucidate the clinical manifestations, treatment, and outcomes of CVT in early pregnancy. To the best of our knowledge, this is the largest and with the longest post-pregnancy follow-up hitherto reported.

## Materials and methods

### Case series

Between May 2018 and November 2021, we retrospectively reviewed the records of all patients hospitalized in the obstetrics and gynecology department of Renji Hospital, School of Medicine, Shanghai Jiao Tong University. A total of 26 hospitalized patients received a diagnosis of pregnancy-associated CVT, and seven patients (26.9%) diagnosed with CVT in early pregnancy (<12 weeks of gestations) were identified and included in this case series. We collected data regarding patients' characteristics, laboratory and neuroimaging findings, treatment modalities, maternal outcomes, and 6-month and 1-year follow-up. Written informed consent was obtained from all the participants before the start of the study. The study was performed in compliance with the Declaration of Helsinki.

## Literature review

We searched the Medline, Embase, and Google Scholar databases to identify all English literature published using the keywords “cerebral venous thrombosis”, “intracranial venous thrombosis”, “first-trimester pregnancy”, “early pregnancy”, alone or in combination. We reviewed all articles (including case reports, case series, and review articles), and this resulted in the inclusion of fourteen case reports with a total of 15 patients (5–18).

## Results

### Study population

We included seven patients in our case series, and all of them were in the first trimester of pregnancy (<12 weeks of gestations). The mean age was 32.4 ± 2.28 years (range 27–42 years), and the mean BMI was 21.67 kg/m^2^ (range 19.5–23.4 kg/m^2^). Five (71%) out of the seven patients were primiparas, and two (N1 and N2) conceived through assisted reproductive technology (ART). None of the patients reported a past or family history of venous thromboembolism (VTE), thrombophilia, or autoimmune or hematologic diseases.

All the patients presented with nausea, vomiting, and various extents of headache. Five of them were initially diagnosed with hyperemesis gravidarum (HG), and the other two presented with mild nausea and vomiting. A visual analog scale (VAS) was used to assess the severity of headache on a scale of 0 to 10, with “0” indicating no pain and “10” indicating worst pain. The headaches described by the patients were mild at the beginning with a mean score of 3.86 and progressed to 7.16 after several days. The mean time from symptom onset to admission was 5.7 days (range 3–10 days). At the time of evaluation in our institution, all the patients presented with acute neurologic symptoms including aphasia (*n* = 5, 71.4%), limb weakness (*n* = 4, 57.1%), seizures (*n* = 2, 28.6%), altered mental status (*n* = 3, 42.9%), and blurred vision (*n* = 2, 28.6%). The Glasgow Coma Scale (GCS) was used to assess the extent of impaired consciousness. One patient (N4) presented as a transfer from an outside hospital due to acute comatose state with a GCS score of 3. Two patients, N6 and N7, had a score of 9 and 11, respectively, consistent with moderate brain injury and presented with various neurological deficits. The remaining four patients (N1, N2, N3, and N5) scored 13 or higher consistent with mild brain injury. A summary of the patients' characteristics is shown in Table 1.

**Table 1:** Characteristics and clinical symptoms of included seven pregnancy cases with CVT.

**Patient**	**Age (years)**	**G/P**	**BMI (kg/m^2^)**	**Gest (week)**	**Personal history**	**Initial symptoms**		**Time from symptom onset to admission (days)**	**Acute neurological symptoms at admission**						
						**NVP/HG**	**VAS of headache**		**VAS of headache**	**Epileptic seizures**	**Limb weakness**	**Aphasia**	**Disturbed consciousness**	**Blurred vision**	**GCS**
N1	42	G1P0	20.70	5	ART	NVP	3	6	8	Yes	Yes	Yes	No	Yes	13
N2	29	G2P0	19.53	7	ART	HG	4	3	7	No	Yes	No	No	No	15
N3	32	G1P0	22.03	11	None	NVP	3	7	6	Yes	Yes	No	No	No	15
N4	33	G5P2	22.10	8	None	HG	5	10	N/A (coma)	No	No	Yes	Yes	No	3
N5	37	G3P1	22.50	8	None	HG	4	7	8	No	No	Yes	No	Yes	13
N6	27	G1P0	21.40	8	None	HG	4	3	7	No	No	Yes	Yes	No	9
N7	27	G1P0	23.40	9	None	HG	4	3	7	No	Yes	Yes	Yes	No	11

G/P, gravidity/parity; Gest, gestational; ART, assisted reproductive technology; NVP, nausea and vomiting of pregnancy; HG, hyperemesis gravidarum; VAS, visual analogue scale; N/A, not available; GCS, Glasgow Coma Scale.

### Laboratory and radiographic findings

As showed in Table 2, six of the total seven patients had elevated WBC count, and all of them, were found to have D-dimer elevation (range from 0.8 to 11.6 mg/L). Serum chemistries, thyroid function, and coagulation studies were normal/non-contributory in all the patients. With regard to thrombophilia testing, only one patient (N3) was found to have a mildly reduced protein S level (43.8%, normal range 50–80%). One patient (N5) was found to have a slightly positive antinuclear antibody (ANA) with a titer of 1:160, but the remaining autoimmune panels were negative. All the patients were ruled out for any autoimmune etiologies after the interdisciplinary evaluation by obstetricians and rheumatologists. In addition, two patients (N1 and N3) with concern of meningitis underwent lumbar puncture, and the results of cerebral fluid (CSF) studies were noncontributory except for elevated opening pressure.

**Table 2:** Major relevant laboratory results of included patients with CVT.

**Patient**	**WBC** (*10^9^/L)	**RBC** (*10^12^/L)	**HB** **(g/L)**	**Hct**	**Platelet** (*10^9^/L)	**PT** **(S)**	**APTT** **(S)**	**D-dimer** **(mg/L)**	**Hcy** **(umol/L)**	**ANA**	**Antsi-dsDNA** **(IU/ml)**	**ACA**	**LAC**	**PS/PC**	**CSF**
N1	10.99	4.68	134	0.407	225	11.3	29.2	0.8	10	N	21.92	N	N	N	N
N2	20.33	4.39	137	0.397	175	10.4	26.1	1.05	8.2	N	24.98	N	N	N	N/A
N3	7.99	3.88	130	0.380	184	12	25.3	1.11	N/A	N	21.74	N	N	Low PS	N
N4	11.70	3.73	110	0.327	182	14.3	33.2	3.8	N/A	N/A	N/A	N/A	N/A	N	N/A
N5	14.55	4.70	137	0.390	267	11.4	25.4	1.6	N/A	1:160	26.45	N	N	N	N/A
N6	24.77	4.61	137	0.379	201	11.7	25.1	8.91	7.9	N	21.57	N	N	N	N/A
N7	10.40	4.60	131	0.390	294	12.2	25.9	0.87	6.7	N	21.22	N	N	N	N/A

WBC, white blood cell; RBC, red blood cell; HB, hemoglobin; Hct, hematocrit; PT, prothrombin time; APTT, activated partial thromboplastin time; Hcy, homocysteine; ANA, antinuclear antibody; anti-dsDNA, anti-double strain DNA; ACA, anticardiolipin antibody; LAC, lupus anticoagulant; PS/PC: protein S/protein C; CSF, cerebrospinal fluid; N/A, not available; N, normal.

Six patients underwent CT, and the findings demonstrated cerebral infarction (*n* = 3, 42.9%), intracranial hemorrhage (*n* = 3, 42.9%), subarachnoid hemorrhage (*n* = 3, 42.9%), and cerebral edema (*n* = 3, 42.9%); also, hyperdense lesions (*n* = 4, 57.1%) were found occasionally in the venous sinus, indicating thrombosis. Six patients underwent magnetic resonance venography (MRV), which demonstrated CVT in all of them. Most of the patients had multiple venous sinus involvement, and the mean number of venous sinuses involved was 3.7 (range 2–6), with the transverse sinus being the most common site (*n* = 7, 100%) followed by the sigmoid sinus (*n* = 5, 71.4%). Neuroimaging features of patients are shown in Table 3.

**Table 3:** Neuroimaging features of the included patients with CVT.

**Patient**	**Imaging modalities**	**Imaging features**					
		**Cerebral infarction**	**Intracranial hemorrhage**	**Subarachnoid hemorrhage**	**Cerebral edema**	**Location of sinuses involvement**	**Cerebral vein thrombosis**
N1	CT, MRI/MRA/MRV	N	Yes	Yes	N	SSS,SS,LTS,RTS,LSS,ISS5	N
N2	CT, MRI/MRA/MRV	N	N	N	N	SSS,RTS,RSS,SS,TH5	N
N3	MRI/MRA/MRV	Yes	N	N	N	SSS,RTS2	Cortical veins
N4	CT, DSA	N	Yes	N	Yes	LTS,LSS,SS3	GCV
N5	CT, MRI/MRA/MRV	Yes	N	Yes	N	LTS,LSS2	N
N6	CT, MRI/MRA/MRV,DSA	Yes	Yes	Yes	Yes	SSS,LTS,LSS,RTS,RSS,SS6	Cortical veins
N7	CT, CTV, MRI/MRA/MRV,DSA	N	N	N	Yes	SS,ISS,LTS,TH4	GCV,ICV

CTV, CT venography; MRI/MRA/MRV, magnetic resonance imaging/magnetic resonance angiography/magnetic resonance venogram; DSA, digital subtraction angiography; SSS, superior sagittal sinus; SS, straight sinus; ISS, inferior sagittal sinus; RTS, right transverse sinus; LTS, left transverse sinus; RSS, right sigmoid sinus; LSS, left sigmoid sinus; TH, torcular herophili; GCV, great cerebral vein; ICV, internal cerebral vein; N, normal.

### Therapy and outcomes

All the patients were started on subcutaneous low-molecular-weight heparin (LMWH) at full anticoagulation doses once the diagnosis of CVT was confirmed. Six patients (85.7%) received osmotic therapy such as mannitol and diuresis to decrease intracranial pressure. Three patients (42.9%) received antiepileptic therapy.

**Figure 1:**
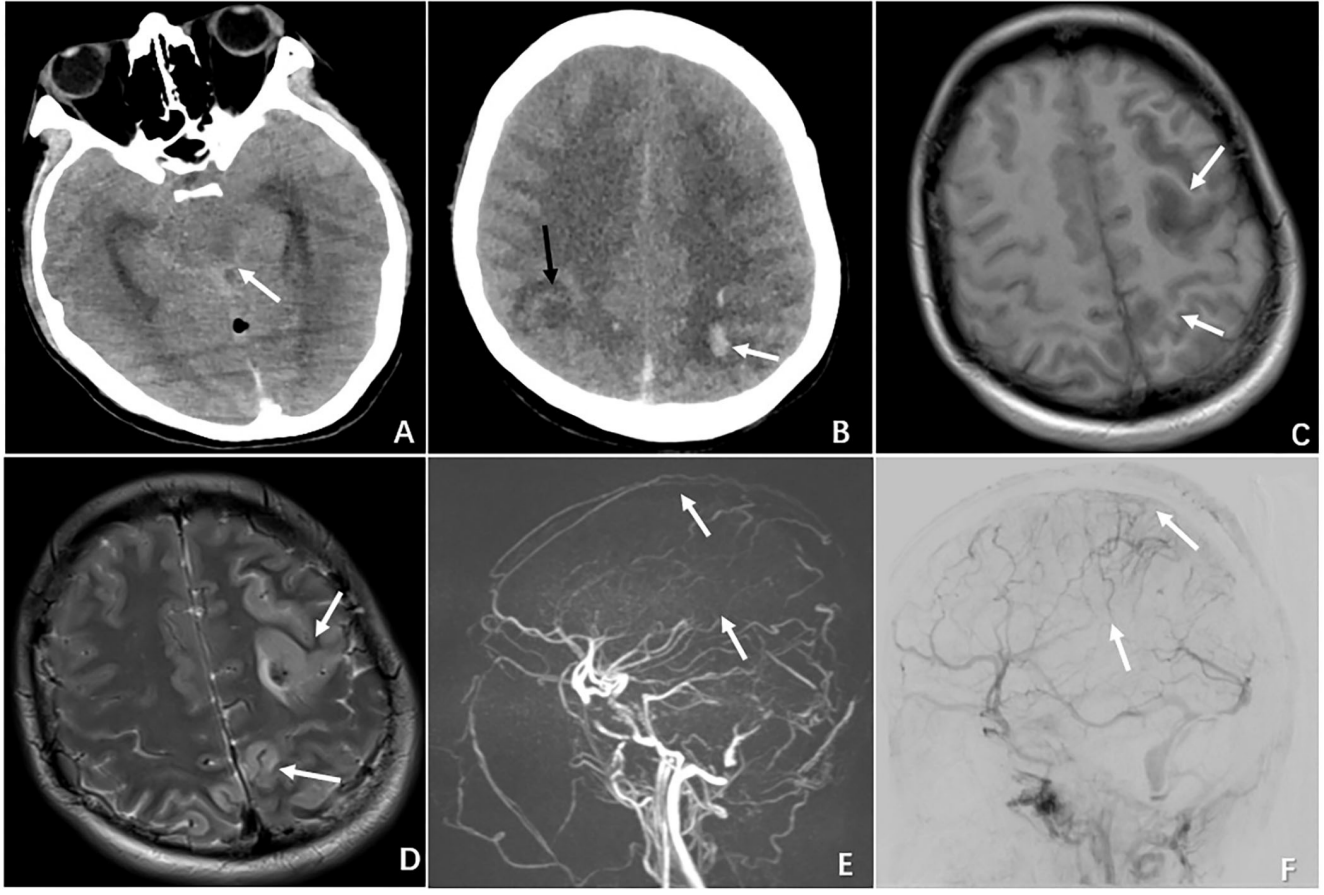
Radiographic features of CVT in the case of N6 who presented with severe neurological deterioration. **(A)** Non-contrast CT demonstrating brain swelling and ambient cistern compression (white arrow). **(B)** CT demonstrating multiple ischemic infarctions (black arrow) with a hemorrhagic component (white arrow). **(C)** MRI showing a left hyperintense lesion with a surrounding hypointense area on T1-weighted MRI. **(D)** MRI demonstrating the same hypointense lesion with a surrounding large hyperintense area on T2-weighted MRI. **(E)** MRV showing complete occlusion of the superior sagittal sinus (superior arrow) and inferior sagittal sinus (inferior arrow). **(F)** DSA demonstrating occlusion of the superior sagittal sinus (superior arrow) and inferior sagittal sinus (inferior arrow).

Three patients with evidence of moderate or severe brain injury proceeded with digital subtraction angiography (DSA), and two underwent endovascular intervention. The treatment course is described below. One patient (N4) presented with a GSC score of 3 (M1V1 E1); the emergent CT revealed cerebellar hemorrhage and brain stem edema, and the subsequent DSA confirmed a complete occlusion in the great cerebral vein, straight sinus, left transverse sinus, and sigmoid sinus. A decision was made to proceed with emergent venous thrombectomy with heparin and urokinase *via* injection through a microcatheter positioned directly to the clot. However, the intervention failed to dissolute the clot and the patient died 4 days after the procedure. One patient (N6) presented with a GCS score of 9 (M4V1 E4) and had radiographic evidence of subarachnoid hemorrhage, left frontal and bilateral parietal lobe hemorrhage complicated by diffuse cerebral edema. The DSA confirmed a complete superficial and deep venous occlusion. Figure 1 demonstrates the radiographic features of CVT in case 6. She underwent emergent venous thrombectomy followed by decompressive craniotomy. Unfortunately, the patient died on postoperative day 4 despite the above measures. One patient (N7) with a GCS score of 11 (M6 V1 E4) underwent DSA, which showed an occluded sinus, but the circulation was compensated by reflux flow from the sylvian vein. Thus, a decision was made to not proceed with endovascular intervention.

Of the 7 patients, two (N4 and N6) died within 5 days because of rapid deterioration. The remaining five patients and their families requested induced abortion; thus, when they were stabilized by the evaluation of a multidisciplinary team, induced abortion with consent proceeded. LMWH was held 12 h prior to the surgery, and the average time from hospitalization to abortion was 6.5 days. The mean length of stay of the surviving patients was 21.4 days. Two patients (N2 and N7) experienced complete recovery and resolution of neurological symptoms on discharge. After pregnancy termination, four patients (N1, N2, N3, and N5) received warfarin, and the doses were strictly adjusted based on an International Normalized Ratio (INR) target of 2–3. The most recently hospitalized patient (N7) received rivaroxaban (20 mg daily) upon discharge.

**Figure 2:**
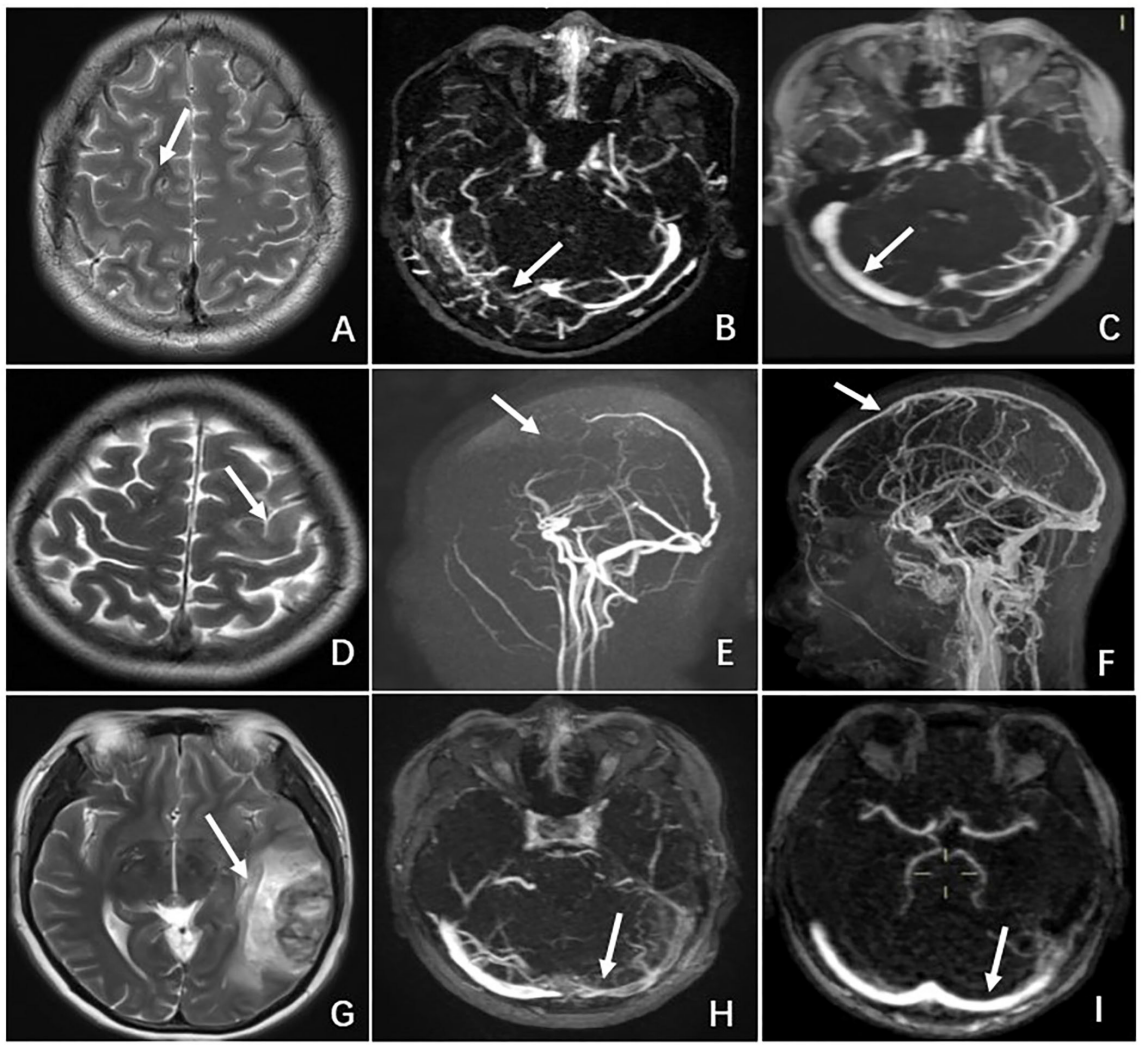
Comparison of radiographic features of CVT between admission and discharge follow-up in 3 cases. Patient N2: **(A)** MRI on admission showing a hyperintense lesion in the right frontal lobe on T2-weighted MRI. **(B)** MRV on admission revealing occlusion of the right transverse sinus. **(C)** MRV on 6-month follow-up demonstrating recanalization of the right transverse sinus. Patient N3. **(D)** MRI on admission showing a hyperintense lesion in the left frontal lobe on T2-weighted MRI. **(E)** MRV on admission revealing occlusion of the superior sagittal sinus and cortical vein. **(F)** MRV on 12-month follow-up demonstrating recanalization of the superior sagittal sinus and cortical vein. Patient N5. **(G)** MRI on admission showing a hyper-and hypo-density lesion in the left temporal lobe on T2-weighted MRI. **(H)** MRV on admission revealing occlusion of the left transverse sinus. **(I)** MRV on 6-month follow-up demonstrating recanalization of the left transverse sinus.

All the patients remained on anticoagulant therapy for 1 year. On the 6-month follow-up, all of them showed complete resolution of neurologic deficits. MRI or MRV was performed either on the 6 or 12-month follow-up visit, and all the patients had radiographic evidence of recanalization of sinuses and/or resolution of thrombi. Table 4 summarizes the treatment and outcomes of the seven patients with CVT. Figure 2 compares the radiographic findings of patients (N2, N3, N5) with CVT on admission and on discharge follow-up.

**Table 4:** Treatment and outcomes of the included patients with CVT.

**Patient**	**Anticoagulation**		**Dehydration**	**AE**	**Endovascular therapy**	**DC**	**Abortion** **after HA** **(days)**	**Pregnancy termination**	**Hosp** **duration** **(days)**	**MRI/MRV before discharge**	**Symptom on discharge**	**Six-month** **FU**	**One-year** **FU**
	**LMWH**	**Bridge to warfarin/ rivaroxaban (days)**											
N1	Yes	14	Yes	Yes	N/A	N/A	9	D&C	22	Partial Reca	Slight Visual ghosting	Asym, Partial Reca	N/A
N2	Yes	7	Yes	N/A	N/A	N/A	4	D&C	20	Partial Reca	Asym	Asym, Partial Reca	Reca
N3	Yes	14	N/A	Yes	N/A	N/A	13	Misoprostol	19	Partial Reca	One limb weakness	Asym	Reca
N4	Yes	N/A	Yes	N/A	Yes	N/A	N/A	N/A	4	N/A	death	N/A	N/A
N5	Yes	17	Yes	N/A	N/A	N/A	5	D&C	22	Partial Reca	Slight Visual ghosting	Asym	Reca
N6	Yes	N/A	Yes	N/A	Yes	Yes	1	D&C	5	N/A	death	N/A	N/A
N7	Yes	14	Yes	Yes	N/A	N/A	7	D&C	18	Partial Reca	Asym	Asym	N/A

AE, antiepileptic; DC, decompressive craniectomy; HA, hospital admission; Hosp, hospitalization; FU, follow-up; D&C, dilatation and curettage; Reca, recanalization; Asym, asymptomatic; N/A, not available.

### Literature review

Our literature review resulted in the inclusion of 15 patients. The first 3 reported cases were diagnosed by postmortem autopsy (two cases in 1957 and one case in 1978), and the remaining 12 cases were diagnosed by neuroimaging. The mean age of the 15 patients was 28.9 ± 2.28 years. Six (40%) out of the 15 patients reported predisposing factors including history of seizure, migraine, antiphospholipid syndrome, oral contraceptive use, and heterozygous factor V Leiden mutation, or history of CVT. All the 15 patients presented with typical neurological symptoms, with headache being the most common symptom (*n* = 12, 80%) followed by seizures (*n* = 6, 40%), altered mental status (*n* = 3, 20%), aphasia (*n* = 2, 13.3%), and hemiparesis (*n* = 2, 13.3%). It is noteworthy that most of the patients also presented with nausea and vomiting of pregnancy (NVP) (*n* = 5, 33.3%) and hyperemesis gravidarum (HG, *n* = 3, 20%). Eleven patients (73.3%) received therapeutic anticoagulation, which included intravenous heparin, subcutaneous LMWH, warfarin, acetylsalicylic acid, and eptifibatide. One patient (6.7%) underwent catheter-directed thrombolysis and thrombectomy. Two patients (13.3%) underwent decompressive craniectomy. Eight patients (53.3%) had complete recovery and resolution of symptoms, while four patients (26.7%) showed residual neurological dysfunction including limb weakness, visual field defect, hemiplegia, and aphasia. Six (40%) patients delivered in the third trimester, and two of them underwent elective cesarean section. All the babies were born healthy. Table 5 summarizes the clinical characteristics and outcomes of the fifteen CVT cases in early pregnancy in the published literature.

**Table 5:** Clinical characteristics and outcome of the 15 included patients with CVT in published literature.

**References**	**Age (years)**	**G/P**	**Gest week**	**Personal history**	**Symptom progression**	**Imaging modality**	**Involved venous**	**Treatment**		**Pregnancy outcome**	**Prognosis**
								**Medical treatment**	**Surgical treatment**		
Fishman et al. (5)	20	N/A	12	N	Dullness→ deep coma	N/A	SSS,SS, TS,TH (necropsy)	N/A	N/A	N/A	Death
Fishman et al. (5)	27	N/A	12	N	NVP,headache→ seizure→ deep coma	N/A	SSS,TH,GCT (necropsy)	N/A	N/A	N/A	Death
Lavin et al. (6)	42	G6P1	8	Seizure	NVP,headache→ seizure	N/A	LTS,RTS, RSS,LSS (necropsy)	N/A	N/A	N/A	Death
Dzialo and Black-Schaffer (7)	24	N/A	12	Migraine	NVP,headache→ dizziness→ seizure→ Limb weakness	CT,MRI	RTS,RSS,SS	Systemic heparin→ intravenous heparin→ enoxaparin	N/A	Delivery	One Limb weakness
Weatherby et al. (8)	29	G2P1	9	N	Headache→ confused	CT, MRI/MRV	SSS	Intravenous heparin→ sub-cutaneous heparin	N/A	Vaginal delivery	Asym
Hanprasertpong et al. (9)	20	G1P0	10	Antiphospholipid syndrome	Headache, hemiparesis.	CT,MRI	SSS,RSS, RTS	LMWH → warfarin	N/A	Vaginal delivery	Asym
Dangal and Thapa (10)	27	G2P0	10	OCT	NVP,headache	CT, MRI/MRV	CVT	LMWH → warfarin	N/A	Abortion	Asym
Munira et al. (11)	34	G3P2	8	N	HG→ headache,diplopia	MRI/MRV	RTS,SSS	Intravenous heparin→ LMWH→ warfarin	N/A	Vaginal delivery	Asym
Yamamoto et al. (12)	32	N/A	9	N	head dullness→ headache,fever→ seizure	CT MRI	SSS,RTS, vein of Galen,SS	Dehydration	DC	Induced abortion	Slight visual field defect
Nie et al. (13)	27	G2P1	5	N	Headache, slurred speech	CT,MRI/MRV,DSA	LTS,LSS	LMWH→ warfarin	N/A	Abortion	Asym
Maeda et al. (14)	35	G5P1	8	N	Seizures	MRI/MRV	SSS,RTS	Intravenous heparin→ LMWH→ warfarin	N/A	Cesarean delivery	Asym
Feng et al. (15)	32	G2P1	10	N	headache→ Seizures→ unconsciousness	CT, MRI/MRV	RTS,RSS	LMWH	N/A	Abortion	Asym
Zhang et al. (16)	22	G1P0	10	Migraine	NVP,headache	CT, MRI/MRV	SSS,RTS	LMWH→ warfarin	N/A	Induced abortion	Asym
Serna Candel et al. (17)	34	G2P1	10	Heterozygous factor V Leiden mutation,CVT	HG,headache→ motor aphasia→ global aphasia	MRI	All venous sinuses and deep internal cerebral veins	LMWH→ ASA→ eptifibatide→ LMWH	Endovascular therapy	Cesarean delivery	Mild neurological deficits
Bertani et al. (18)	28	N/A	9	N	HG→ confusion, and impaired balance.	CT	RTS,LTS	Heparin→ oral rivaroxaban	DC	Abortion	Hemiplegia and aphasia

G/P, Gravidity/Parity; Gest, Gestational; NVP, Nausea and Vomiting of Pregnancy; HG, Hyperemesis Gravidarum; DC: Decompressive craniectomy; OCT, oral contraceptive. SSS, superior sagittal sinus; SS, straight sinus; ISS, Inferior sagittal sinus; RTS, Right Transverse sinus; LTS, left transverse sinus; RSS, right Sigmoid sinus; LSS, left Sigmoid sinus; TH, torcular Herophili; GCT, Great cerebral vein; N/A, not available.

## Discussion

CVT is a rare case of pregnancy-related stroke, especially in early pregnancy. In the present study, we reported a retrospective case series of CVT presenting in early pregnancy complicated by nausea and vomiting in pregnancy/hyperemesis gravidarum (NVP/HG). NVP is a common condition with prevalence rates of up to 50–80% for nausea and 50% for vomiting and retching (19). The Royal College of Obstetricians and Gynecologists' (RCOG) green-top guidelines suggest that NVP/HG is a risk factor for VTE, as it often results in dehydration, malnutrition, and anemia, leading to hemoconcentration (20). Fiaschi L et al. reported a cohort study on 82,11,850 pregnancies resulting in live births or stillbirths, and women with HG had increased odds of antenatal VTE, including deep vein thrombosis (OR 1.94, 99% CI 1.57, 2.39) and pulmonary embolism (OR 2.54, 99% CI 1.89, 3.4) (21).

The pathogenesis of CVT remains to be incompletely understood, but two predominant mechanisms that may contribute to the clinical features of CVT have been proposed: (1) thrombosis of sinus results in increase in venular and capillary pressure leading to serial parenchymal insults including ischemia, edema, and hemorrhage; (2) occlusion of cerebral veins results in impaired CSF absorption leading to elevated intracranial pressure. Headache, generally indicative of an increase in intracranial pressure, is the most common symptom and presents in nearly 90% of patients with CVT (22). In our case series, all patients presented with nausea, vomiting, and various extent of headache with an average from 3.86 to 7.16 score by VAS over several days. However, headache is nonspecific and is often considered a common presentation of NVP/HG; as a result, a diagnosis of CVT is often overlooked initially in the absence of other neurological symptoms. In addition, as shown in the literature review of the 15 patients, NVP was found in 5 patients and HG was found in 2 patients, with headache being the most common symptom (*n* = 12, 80%). The multivariate analysis in a systematic review demonstrated that headache alone was associated with favorable outcome for CVT in pregnancy and puerperium (*p* = 0.04), and that coma/obtundation was associated with worse outcome (*p* = 0.03) (23). Likewise, our results indicated that low GCS score on admission may predict poor prognosis for patients with CVT in early pregnancy. Thus, clinicians should be prudent that persistent or aggravating headaches in patients with NVP/HG could be an initial sign of CVT, and that early diagnosis and prompt treatment are important. Delay in diagnosis can lead to irreversible and life-threatening neurological sequelae, as shown in our case series.

The mainstay of treatment for CVT is anticoagulation with intravenous heparin or subcutaneous LMWH. In our case series, all the patients received subcutaneous LMWH with full anticoagulation doses once the diagnosis was confirmed. Warfarin and new oral anticoagulants (NOAC) are contraindicated in pregnancy because of potential teratogenic effects and risk of fetal hemorrhage (24). There is no definitive evidence regarding the optimal duration of anticoagulant therapy. In our case series, 5 patients remained on anticoagulant therapy for 1 year to reduce the risk of recurrent CVT: 4 patients received warfarin with an INR goal of 2–3, and the most recently hospitalized patient received rivaroxaban (20 mg daily). No adverse effects from the anticoagulant therapy were reported, and all the patients showed radiographic evidence of recanalization or thrombus resolution on the 12-month follow-up.

Endovascular treatment of CVT remains controversial and is usually reserved for patients who are comatose or deteriorating despite anticoagulation. A systemic review of 183 cases of CVT undergoing mechanical thrombectomy revealed that 40% of the patients were in a comatose state at the time of the procedure. Following mechanical thrombectomy, 9% of the patients had worsening or new intracranial hemorrhage, 69% had complete recanalization, and 35% had complete recovery (25). Decompressive craniectomy has shown to be beneficial to critically ill patients with radiological and clinical features of mass effect, signs of brainstem dysfunction, and/or refractory intracranial hypertension (26). However, evidence regarding pregnant or puerperal populations is limited. In our case series, venous thrombectomy was performed on two patients, both experienced rapid deterioration and died. One patient subsequently proceeded with decompressive craniotomy.

We do not have enough evidence to provide the fetal outcomes of CVT in early pregnancy in our study. A shared decision was made after a thorough discussion of the diagnoses, treatment options, maternal and fetal outcomes, and all the five patients decided to proceed with pregnancy termination. Based on the literature review, 6 (40%) out of the 15 patients delivered in the third trimester and all the babies were born healthy. We believe that CVT is not an absolute indication for pregnancy termination, and it has been reported that in women with prior cerebral venous thrombosis, recurrent venous thrombotic events during subsequent pregnancies are infrequent (27, 28). Thus, the decision-making on continuing or terminating a pregnancy should be a shared process and based on the patient's ideologies. A multidisciplinary team of specialists with expertise should be involved in management of CVT in early pregnancy given the complexity and acuity of its nature.

## Conclusion

In summary, CVT in early pregnancy represents a diagnostic challenge given its rarity and nonspecific overlapping clinical features with HG, which could lead to delay in diagnosis and result in rapid deterioration. Urgent MRI/MRV remains the cornerstone for diagnosis, and anticoagulation is the mainstay of treatment. CVT in early pregnancy is not an absolute indication for pregnancy termination. A multidisciplinary team of specialists with expertise should be involved in management of CVT in early pregnancy. Larger patient series with a longer follow-up are warranted to draw more definitive conclusions on the subject.

## Data availability statement

The original contributions presented in the study are included in the article/supplementary material, further inquiries can be directed to the corresponding authors.

## Ethics statement

Ethical review and approval was not required for the study on human participants in accordance with the local legislation and institutional requirements. Written informed consent from the patients or patients legal guardian/next of kin was not required to participate in this study in accordance with the national legislation and the institutional requirements.

## Author contributions

CW, JL, and NZ conceived and designed the study and interpreted the results. CW collected and analyzed the data. XH: figure preparation. CW and KL contributed to the writing of the manuscript. All authors contributed to the article, read, and approved the final version of the manuscript.

## Funding

This study was supported by the Natural Science Foundation of Science and Technology Commission of Shanghai Municipality (No. 22ZR1438700).

## Conflict of interest

The authors declare that the research was conducted in the absence of any commercial or financial relationships that could be construed as a potential conflict of interest.

## Publisher's note

All claims expressed in this article are solely those of the authors and do not necessarily represent those of their affiliated organizations, or those of the publisher, the editors and the reviewers. Any product that may be evaluated in this article, or claim that may be made by its manufacturer, is not guaranteed or endorsed by the publisher.

## Abbreviations

ART: assisted reproductive technology
CVT: cerebral venous thrombosis
DSA: digital subtraction angiography
VAS: visual analog scale
GCS: glasgow coma scale
HG: hyperemesis gravidarum
LMWH: low-molecular-weight heparin
MRI: magnetic resonance imaging
MRV: magnetic resonance venography
VTE: venous thromboembolism.
